# Routine incorporation of longer-term patient-reported outcomes into a Dutch trauma registry

**DOI:** 10.1007/s11136-019-02211-y

**Published:** 2019-05-16

**Authors:** Quirine M. J. van der Vliet, Abhiram R. Bhashyam, Falco Hietbrink, R. Marijn Houwert, F. Cumhur Öner, Luke P. H. Leenen

**Affiliations:** 1 University Medical Center Utrecht, Heidelberglaan 100, 3508 GA Utrecht, The Netherlands; 2 Department of Surgery, University Medical Center Utrecht, Heidelberglaan 100, 3508 GA Utrecht, The Netherlands; 3 Harvard Combined Orthopaedic Residency Program Boston, Boston, USA

**Keywords:** Patient-reported outcomes, Trauma, Trauma registry, Benchmarking, Quality improvement

## Abstract

**Purpose:**

Routine collection of post-discharge patient-reported outcomes within trauma registries can be used to benchmark quality of trauma care. This process is dependent on geographic and cultural context, but results are lacking regarding the European experience. We aimed to investigate the feasibility of routine inclusion of longer-term patient-reported health-related quality of life (HRQoL) in a Dutch National Trauma Database (DNTD) and to characterize these outcomes in a prospective cohort study.

**Methods:**

All adult patients (≥ 18 years) who presented for traumatic injury in 2015–2016 and met the inclusion criteria of the DNTD were included. Inclusion criteria of the DNTD are presence of traumatic injury, hospital presentation within 48 h from trauma and hospital admission for treatment of traumatic injury or immediate mortality from traumatic injury after presentation. Exclusion criteria were death, mental impairment, insufficient command of Dutch language and residency outside the Netherlands. Primary outcomes were process-related measures of feasibility (response rate, response methods and reasons for non-response). Secondary outcomes were HRQoL measures [EuroQOL 5-Dimensions 3-Level (EQ-5D-3L) with added cognitive dimension and Visual Analogue Scale (EQ-VAS)].

**Results:**

2025 unique patients met the initial inclusion criteria, with 1753 patients eligible for follow-up. Of these, 1315 patients participated (response rate 75%). The majority of questionnaires, 990 (75%), were completed on paper, with an additional 325 (25%) through telephone interviews. Primary reason for non-response was lack of contact information (245/438 non-responders; 56%). Median EQ-5D score was 0.81 (IQR 0.68–1.00) (mean 0.74; SD 0.31) and median EQ-VAS score was 78 (IQR 65–90). Compared to a Dutch reference population (mean EQ-5D = 0.87), EQ-5D scores were significantly lower (*p *< 0.001).

**Conclusions:**

Routine collection of HRQoL is feasible within European health systems, like in the Netherlands. Further integration of these measures into trauma registries may aid worldwide benchmarking of trauma care quality.

## Introduction

Trauma registries have been created in many countries to collect data for quality assessment and improvement of trauma care at the regional and national level [1, 2]. Historically, the primary outcome tracked by these registries has been in-hospital mortality. However, as trauma care improved, in-hospital mortality markedly decreased and its use as the sole patient outcome variable is no longer sufficient [2–5]. As a result, trauma registries evolved to incorporate functional status at discharge, but studies have since shown that outcomes collected at discharge do not predict the long-term outcomes valued by patients (i.e. disability and quality of life) [6, 7].

Given that the vast majority of trauma patients survive their injuries, the most recent iterations of trauma registries have started to incorporate post-discharge, longer-term patient-reported outcomes (PROs). Incorporating these outcomes into existing trauma registries is necessary for benchmarking institutions, informing clinical decision-making, and evaluating and improving overall quality of trauma care [8, 9]. In fact, it is now well accepted that systematic outcome measurement is essential for value improvement, the ultimate goal of trauma registries [2, 10].

Routine, population-based follow-up of adult trauma survivors following hospital discharge was first successfully reported in the Australian Victorian State Trauma Registry (VTSR) in 2006 [11]. The data from the VTSR has proven to be a valuable resource for international benchmarking of trauma care and for research into post-trauma PROs, with various studies and improvement projects resulting from this data [12–15]. As a result, the methodology of the VTSR has now been adapted and investigated for use with trauma registries in the United States, Hong Kong, and New Zealand [8, 16, 17]. Yet, two recent studies have suggested that routinely including PROs into trauma registries can vary by cultural context. The FORTE project in the United States demonstrated that incorporation of PROs was feasible, but that better methods for collection of the data were needed because of low response rates, even when using the original VTSR methodology with additional financial incentives [8]. Similarly, implementation of routine post-discharge PROs collection for trauma patients in Hong Kong demonstrated differences in response rate and outcomes compared to patients in Australia, with Australians responding more often and reporting better outcomes without adjusting for confounders [13]. Comparable data is unavailable for Europe, as there are no reports of prospective routine collection of post-discharge PROs in any European country [7–9, 11].

Given the importance of geographical and cultural context emphasized by prior work, we investigated the feasibility of routine inclusion of longer-term patient-reported health-related quality of life in the Dutch National Trauma Database (DNTD) using process-related outcomes of feasibility (response rate, response methods, workload, and reasons for non-response). Our project was also motivated by the fact that the Dutch Ministry of Health commissioned the National Health Care Institute to carry out the Outcome Information for Joint Decisions program, a program framed to collect outcome information on 50% of the complete burden of disease in the Netherlands [18]. As trauma-related injuries account for a substantial part of this burden, the Dutch National Trauma Database (DNTD) provides an important target area [19]. We hypothesized that incorporating PROs into the DNTD was possible. As a secondary objective, we sought to characterize the longer-term outcomes of Dutch trauma patients after injury.

## Methods

### Data sources and patient population

#### Study design

Prospective cohort study.

#### Setting

The University Medical Center Utrecht is a Joint Commission International (JCI) accredited tertiary care facility with 1000 beds, complying with all requirements defined by the American College of Surgeons’ Committee on Trauma [20]. This center is the designated level 1 trauma center of an inclusive trauma system situated in the Central Netherlands region, serving approximately 1.2 million people in collaboration with nine level II and III centers [21].

#### Patient population

**Fig. 1:**
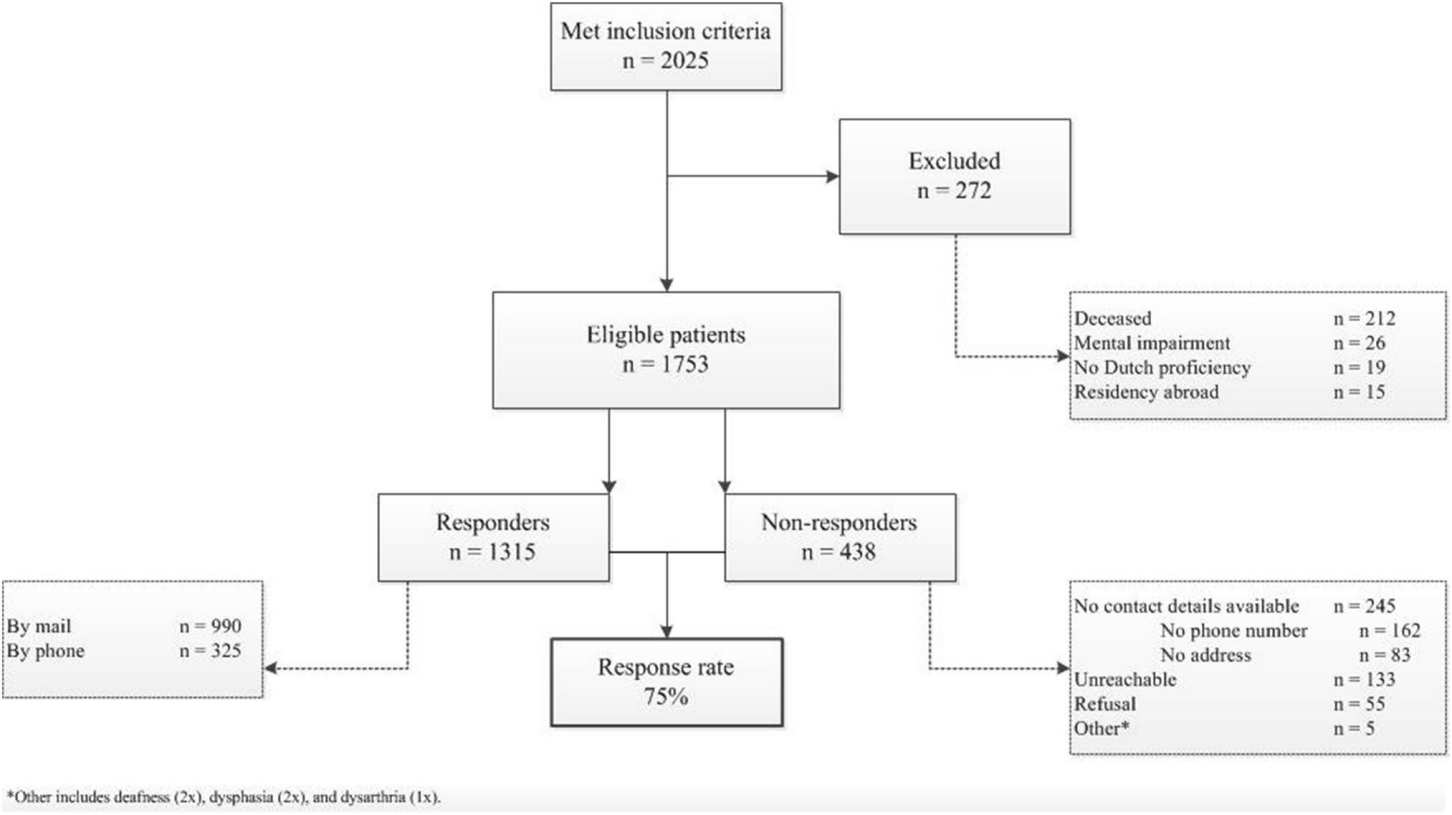
Flowchart of inclusion and response process

Eligible patients were identified from the Dutch National Trauma Database (DNTD). The DNTD is a prospectively collected database of all admitted trauma patients, continuously monitored by trained data managers and trauma surgeons. Adult patients (≥ 18 years) who presented to our institution for assessment of traumatic injury between January 1, 2015 and December 31, 2016, and met the inclusion criteria of the DNTD were included. Inclusion criteria of the DNTD are presence of traumatic injury, hospital presentation within 48 h from trauma and hospital admission for treatment of traumatic injury or immediate mortality from traumatic injury after presentation. Deceased patients, mentally impaired patients, patients with insufficient command of Dutch language and patients residing outside the Netherlands were excluded (Fig. 1). The vital status was assessed using the municipal personal records database. Mental impairment and language proficiency were based on chart reviews as well as telephone interviews in case of uncertainty.

Patients who were eligible for follow-up were sent a recruitment letter explaining the healthcare evaluation together with a questionnaire assessing health-related quality of life (HRQoL), and a stamped return envelope. Letters were mailed to the home addresses listed in the electronic medical record (EMR). Patients with multiple trauma admissions during the current study period were approached only once. Recruitment letters were sent in batches four times a year; patients were included in a given batch if they were within approximately 3 months of 1 year post-injury. If no response was received within 3 months after sending out the initial letter, a reminder letter was sent. The process of sending recruitment and reminder letters was performed by a trained data manager and a secretary. In case of non-response after both letters, patients were contacted by telephone for verbal administration of the questionnaire. Interviews were carried out by a trained medical student and a member of the research team (QV). The timing of the telephone calls was varied in an attempt to maximize response rate [8]. After three failed telephone calls, a patient was deemed a non-responder.

### Outcome measures

Primary outcomes were process-related measures of feasibility (response rate, response methods, workload, and reasons for non-response). Response rate was defined as the number of patients that responded to the questionnaire divided by the total amount of patients eligible for follow-up. The total workload needed to incorporate PROMs in the registry, including sending out letters and collecting responses, was estimated using the unit time estimate derived from time-driven activity-based costing methods [22]. Secondary outcomes were HRQoL measures (EuroQOL 5-Dimensions 3-Level (EQ-5D-3L) with an added cognitive dimension (EQ-6D-3L) and EuroQOL Visual Analogue Scale (EQ-VAS)) [23]. The EQ-5D-3L covers five dimensions (mobility, self-care, usual activities, pain/discomfort, anxiety/depression, and cognition), with three possible levels for all dimensions (no problems, some problems, extreme problems) [24]. The EQ-5D scores for the study population were calculated using a scoring algorithm appropriate for a population of Dutch patients, not taking into account the sixth dimension, with a possible range from − 00.33 to 1.00 [25]. The additional sixth dimension enables a description of the cognitive abilities, covering memory, concentration and coherence [23, 26]. It was chosen to evaluate this dimension as trauma is known to carry the potential of impacting cognition, for example due to development of post-traumatic stress disorder [27, 28]. The EQ-VAS is an instrument developed for recording an individual’s current self-rated health on a scale from 0 to 100. For both the EQ-5D and the EQ-VAS, higher scores represent higher health-related quality of life [29].

### Explanatory variables

Data on patient demographics, Injury Severity Score (ISS), and injury locations were obtained from the DNTD. The ISS calculates injury severity based on the most severe injuries sustained per body region, with higher scores indicating more severe injury [30]. Injuries were categorized as serious head, thoracic, or abdominal injury if the Abbreviated Injury Scale (AIS) was greater than 2 in the respective regions. Minor injuries to the extremities were excluded by only including upper or lower extremity injury with AIS > 1.

### Statistical analyses

Responses were collected into a Microsoft Office Excel 2010 database. Descriptive statistics were calculated. Categorical variables were reported as numbers with percentages. Continuous variables were reported as medians with interquartile ranges (IQR) after applying Shapiro–Wilk normality tests. Bivariate analyses using Chi squared and Mann–Whitney *U* tests were performed to compare baseline characteristics between responders and non-responders. Multivariable linear regression analyses were conducted to identify demographic and injury-related factors associated with EQ-5D scores. EQ-5D scores of the study population were compared to the norms for the general Dutch population (0.87) as well as an age-adjusted Dutch reference population (0.86) using the Student’s *t* test [31]. All statistical analyses were performed using STATA^®^ 13.1 (StataCorp LP, TX, USA). A *p* value of < 0.05 was considered statistically significant. This project was approved by the University Medical Center Utrecht Institutional Review Board.

## Results

A total of 2025 unique patients met the initial inclusion criteria. Subsequently, 272 patients were excluded as they were found to be deceased (212/272), mentally impaired (26/272), lacked Dutch language proficiency (19/272), or resided outside of the Netherlands (15/272), resulting in 1753 patients eligible for follow-up. Within this cohort of eligible patients, 1315 patients responded, leading to a response rate of 75%. Median follow-up after the index trauma was 1.6 years (IQR 1.4–2.0, range 1.0–3.0). Figure 1 describes the inclusion and response process of patients. Table 1 describes participants’ demographic and clinical characteristics based on inclusion–exclusion criteria.

**Table 1:** Demographic and clinical characteristics

	Total cohort (*n* = 2025)	Eligible patients (*n* = 1753)	Ineligible patients (*n* = 272)
	Median (IQR)	Median (IQR)	Median (IQR)
Age at injury (years)	54 (34–70)	52 (33–66)	71 (54–83)
Injury severity score (*n* = 1960)	9 (5–17)	9 (5–16)	10 (5–25)

	*n* (%)	*n* (%)	*n* (%)
Male gender	1336 (66)	1176 (67)	160 (59)
Serious^a^ head injury	544 (27)	430 (25)	116 (43)
Serious^a^ thoracic injury	366 (18)	312 (18)	57 (21)
Serious^a^ abdominal injury	67 (3)	57 (3)	11 (4)
Extremity injury	878 (43)	763 (44)	113 (42)
Upper extremity	473 (23)	410 (23)	60 (22)
Lower extremity	543 (27)	471 (27)	68 (25)

*IQR* interquartile range, *n* number

^a^Serious injury was defined as an abbreviated injury scale > 2

The majority of questionnaires, 990 (75% of 1315 responders), were completed on paper, with an additional 325 (25%) completed through telephone interviews. After sending the initial recruitment letters, questionnaires were returned by 802 patients (802/990 mail responses; 81%). Sending reminder letters yielded 188 additional returned questionnaires. Of the 325 patients that completed the questionnaire over the telephone, 197 patients were successfully reached after the first attempt (60% of 325), 80 patients were successfully reached after the second attempt (25%), and 41 patients were successfully reached after the third attempt (13%). A record of the number of attempts was missing for 8 patients so they were unclassified (2%).

It was estimated that approximately 620 working hours per year, comparable to 0.3 to 0.5 full time equivalent (FTE) employee (adjusting for up to 20% error in estimates), was needed to complete the entire process (including identifying eligible patients, sending out recruitment and reminder letters, contacting patients by telephone, data processing, auditing, and monitoring).

The primary reason for non-response was lack of current contact information (245/438 non-responders; 56%). Another 133 patients could not be reached after sending two letters and following three phone call attempts (133/438; 30%). Only 55 patients (13%) were reached but refused to participate (Fig. 1).

Table 2 compares the demographic and clinical characteristics of responders and non-responders. These groups were similar in most characteristics, but, on average, responders were of older age (*p* < 0.001) and had higher ISS score (*p* < 0.001) than non-responders.

**Table 2:** Demographic and clinical characteristics of responders versus non-responders

	Responders (*n* = 1315)	Non-responders (*n* = 438)	*p* value
	Median (IQR)	Median (IQR)	
Age at injury (years)	55 (37–69)	39 (27–56)^a^	**< 0.001**
Injury severity score (*n* = 1960)	9 (5–17)	9 (4–14)^a^	**< 0.001**

	*n* (%)	*n* (%)	
Male gender	879 (67)	297 (68)	0.71
Serious^a^ head injury	332 (25)	96 (22)	0.34
Serious^a^ thoracic injury	241 (18)	68 (16)	0.57
Serious^a^ abdominal injury	43 (3)	13 (3)	0.94
Extremity injury	570 (43)	195 (45)	0.86
Upper extremity	318 (24)	95 (22)	0.17
Lower extremity	351 (27)	124 (28)	0.51

Bold indicates statistically significant difference

*IQR* interquartile range, *n* number

^a^Serious injury was defined as an abbreviated injury scale > 2

Median EQ-5D score was 0.81 (IQR 0.68–1.00; range − 0.33 to 1.00), mean EQ-5D score was 0.74 (SD 0.31) and median EQ-VAS score was 78 (IQR 65–90; range 0–100). EQ-6D composite and component scores are summarized in Table 2. When compared to a Dutch reference population (EQ-5D = 0.87) and an age-adjusted Dutch reference population (EQ-5D = 0.86), EQ-5D scores in our population were significantly lower (*p *< 0.001). For the cognitive dimension, 791 patients (62%) reported no problems, 381 (30%) some problems, and 106 (8%) severe problems.

Using multivariable linear regression analyses, factors independently associated with worse HRQoL (EQ-5D score) were: older age at injury (coefficient (adjusted mean difference) [95% CI] − 0.002 [− 0.003, − 0.001]; *p *< 0.001), higher ISS (coefficient (adjusted mean difference) [95% CI] − 0.004 [− 0.006, − 0.001]; *p* 0.002), female gender (coefficient (adjusted mean difference) [95% CI] − 0.057 [− 0.093, − 0.020]; *p* 0.003), and lower extremity injury (coefficient (adjusted mean difference) [95% CI] − 0.071 [− 0.109, − 0.032]; *p *< 0.001) (Table 3).

**Table 3:** Outcome scores of patients

	Median	IQR	Range
EQ-5D (*n* = 1298)	0.81	0.68–1.00	− 0.33 to 1.00
EQ-VAS (*n* = 1273)	78	65–90	0–100

	*n*	%
Mobility (*n* = 1308)		
No problems	820	63
Some problems	329	25
Extreme problems	159	12
Self-care (*n* = 1309)		
No problems	1092	83
Some problems	159	12
Extreme problems	58	4
Usual activities (*n* = 1310)		
No problems	701	54
Some problems	501	38
Extreme problems	108	8
Pain/discomfort (*n* = 1308)		
No problems	629	48
Some problems	582	45
Extreme problems	97	7
Anxiety/depression (*n* = 1307)		
No problems	935	72
Some problems	306	23
Extreme problems	66	5
Cognition (*n* = 1278)		
No problems	791	62
Some problems	381	30
Extreme problems	106	8

*IQR* interquartile range, *n* number, *EQ-5D* EuroQOL 5-dimensions, *EQ-VAS* EuroQOL visual analogue scale

## Discussion

Our study indicates that routine inclusion of longer-term patient-reported outcomes in a Dutch trauma registry is feasible, with a high response rate and little non-response bias (due to a high response rate and few differences between responders and non-responders). Approximately, 1 year after the traumatic event, health-related quality of life of the patients was moderately inferior compared to the general Dutch population, but was similar to outcomes reported from outside Europe [8, 9, 13]. Factors independently associated with worse longer-term outcome were older age, higher ISS, female gender, and lower extremity injury (Table 4).

**Table 4:** Regression analyses

	Regression coefficient (= adjusted mean difference)^a^	95% CI	*p* value
Age at injury	− 0.002	− 0.003 to − 0.001	**< 0.001**
Injury severity score	− 0.004	− 0.006 to − 0.001	**0.002**
Interval between injury and questionnaire	− 0.030	− 0.066 to 0.007	0.109
Male gender	0.057	0.020–0.093	**0.003**
Serious^b^ head injury	0.004	− 0.038 to 0.047	0.838
Serious^b^ thoracic injury	0.017	− 0.029 to 0.063	0.460
Serious^b^ abdominal injury	0.038	− 0.057 to 0.133	0.435
Upper extremity injury	0.016	− 0.024 to 0.055	0.441
Lower extremity injury	− 0.071	− 0.109 to − 0.032	**< 0.001**
Model	Multivariable linear		

*CI* confidence interval

^a^Positive regression coefficients denote higher EuroQOL 5-dimensions (EQ-5D) scores

^b^Serious injury was defined as an abbreviated injury scale > 2

Many prior projects have attempted to assess patient outcomes after a variety of injuries and treatments by focusing on a narrowly defined subset of the population at a particular time period [9, 32, 33]. However, a drawback to this approach is that information pertaining to patients and health systems is limited by study design [34, 35]. This is especially true for trauma patients and trauma systems where it is not uncommon to have high refusal rates and significant follow-up disparity [9, 34, 36, 37]. As a result, these study designs are not sufficient to evaluate or benchmark the quality of care at the institutional, regional, or national level. Thus, recent studies have emphasized the routine inclusion of PROs and HRQoL metrics within trauma registries [8, 13, 16]. This framework allows for the collection of comprehensive, normative data on the long(er)-term outcomes of patients who sustain trauma, summarizes the care received within health systems, and meets regulatory requests for data collection that can facilitate quality improvement, cost-effectiveness, resource utilization and benchmarking studies.

When we adopted this framework in our health system, we found that routine inclusion of HRQoL in a Dutch trauma registry was feasible, with high follow-up percentage and low response bias. Prospective collection of outcome data likely led to much higher response rates compared to retrospective collection. We also found that outcomes approximately 1 year after injury were lower compared to the Dutch reference population [38]. Unlike similar studies in the United States or Hong Kong, only a small percentage of patients refused to participate when asked to respond by paper or telephone questionnaire, even when no additional incentive was provided. This finding highlights another scenario where different geographic and cultural context led to variation in practical implementation of the same adapted protocol [8, 13, 16]. Patients within the Netherlands may have been more eager to participate to share information on their health status and thereby contribute to quality improvement programs. Similar to the problems encountered in the efforts of incorporating PROs into Australian and North American trauma registries, the main reason for non-response in our population was the lack of current contact details [8, 9]. Targeting this problem may lead to a higher response rate. Hospitals should be encouraged to document and verify patient contact information, and patients should be instructed to inform the hospital of changes to contact information. With the advent of EMRs, automatic alerts for providers and staff can be incorporated into the EMR to highlight when a patient has missing or inadequate contact details.

In our study, the majority of questionnaires were completed on paper, and an additional third were administered successfully over the phone. While this led to satisfactory response rates in our setting, future work should investigate the use of other administration modalities, especially since the majority of non-responders were younger patients who may use email or smartphone applications as their primary communication tools [39]. Currently, we are exploring the use of a web-based platform for the distribution of patient-reported outcome measures (PROMs).

Responders and non-responders differed significantly with respect to age and ISS. Similar age-related differences between responders and non-responders have been identified previously [8, 9]. In our catchment area, many young patients may be students who move more frequently and whose contact information changes more often. Higher ISS in responders has also been documented in the Victorian State Trauma Registry [9]. As ISS was independently associated with worse quality of life, results of the present study as well as previous studies may underestimate HRQoL in the complete trauma population. A potential explanation for the differences in injury severity is that patients who suffer less severe trauma, with higher odds of returning to pre-injury health status, are less inclined to document their quality of life as they are not confronted with the consequences of their injuries on a daily basis. Differences between responders and non-responders affect generalizability of studies measuring outcomes after trauma, and our study found patterns similar to those observed in other countries [37].

Due to the unexpected nature of trauma, pre-injury baseline information about patients is often either missing or subject to patient recall bias. This can make it challenging to counsel patients on average expected outcomes or to benchmark across different trauma populations [13]. By routinely incorporating outcomes into trauma registries for all patients, results on average converge to expectations for the general population as the sample size grows. Statistically, this information can then be used to counsel patients and provide benchmarking information for a cohort of patients on average [40].

Even though routine inclusion of patient-reported outcomes in a Dutch trauma registry was deemed feasible, our results were only accomplished with major time investment. Sending out letters, processing returns and conducting telephone interviews created an important time burden for our data managers (0.3–0.5 FTE). In order for the standard incorporation of PROs to be sustainable, additional technological and financial support and manpower are required. With trauma registries being established to improve care, their contents should be curated to allow for accurate, updated data.

Patients included in our study reported lower HRQoL in comparison to the Dutch reference population and a substantial amount of patients suffered from cognitive impairment. We identified factors associated with worse outcomes. Future study should focus on causative relations between trauma and the injuries sustained and longer-term HRQoL. In addition, it should be investigated whether comparisons between a trauma population and the general population are actually valid, as studies suggest that trauma patients may not be completely representative of the general population [41–43].

Our study has several limitations. First, the study population was limited to one level 1, tertiary referral center that typically serves severely injured patients and patients with significant comorbidities, which may not be representative of the whole trauma population. Further validation in other settings (especially the complete Dutch Trauma Registry) will be important, but inclusion of functional outcomes that can be compared to population norms should improve generalizability [8]. Second, for some patients, telephone interviews were performed for more than 1 year after trauma as the protocol for phone interviews was added to the initial protocol to boost response rates. Therefore, in our regression analyses, we adjusted for the interval between trauma and completion of the questionnaire. Third, as there is lack of tailored instruments for trauma patients and no consensus on the most optimal tool, making comparisons with similar projects and registries is difficult. We chose to use the EQ-5D with added cognitive dimension and EQ-VAS as they are questionnaires that can discriminate between a wide variety of health states [29]. Keeping our questionnaire simple and short is one reason we believe that our response rate was high. Fourth, we chose to exclude patients that were mentally impaired or had no proficiency of the Dutch language. This may have introduced bias in our results, especially since patients with severe traumatic brain injury could have been excluded. However, as the number of patients that were excluded based on these criteria was low, we believe the effects to be minimal. Nonetheless, future study should be directed towards the use of proxy questionnaires and non-Dutch versions of the questionnaires for outcome evaluation in a Dutch trauma population. Fifth, two modalities of questionnaire administration were used. This may have influenced the outcomes reported. However, the EQ-5D has been developed to be administered both on paper and verbally and we used the paper version and telephone script according to the EuroQOL instructions [24].

Routine collection of HRQoL is feasible within European health systems, like in the Netherlands. Paper and telephone questionnaire methods have high response rate even when no incentive is provided, but lack of response among younger patients highlights the importance of accurate contact information and exploration of electronic communication modalities. In addition, collection of this data demonstrates that trauma patients in the Netherlands continue to report some impairment compared to the general population approximately 1 year following injury. Further integration of PROs and HRQoL into trauma registries may aid with worldwide benchmarking of the quality of trauma care and decrease the gap in our understanding of the impact of trauma on both patients and society.
